# Ciliated Hepatic Foregut Cyst: Definitive Diagnosis Is Critical to the Optimal Treatment Pathway

**DOI:** 10.1155/2023/6637890

**Published:** 2023-07-19

**Authors:** Tatsuhiro Kato, Christine M. G. Schammel, Hubert Fenton, Steven D. Trocha, A. Michael Devane

**Affiliations:** ^1^University of South Carolina School of Medicine Greenville, Greenville, SC 29605, USA; ^2^Pathology Associates, Greenville, SC 29605, USA; ^3^Department of Surgery, Prisma Health Upstate, Greenville, SC 29605, USA; ^4^Department of Radiology, Prisma Health Upstate, Greenville, SC 29605, USA

## Abstract

*Background*. Ciliated hepatic foregut cyst (CHFC) is a rare, benign cyst of the liver, derived from the embryonic foregut epithelium. Although CHFCs are typically asymptomatic, some present with nonspecific abdominal symptoms. Imaging modalities alone are insufficient for diagnosis, with intrahepatic cholangiocarcinoma included in the differential due to nonspecific imaging features; definitive diagnosis relies on histologic confirmation. These lesions are often benign; however, larger lesions can have malignant transformation into squamous cell carcinoma (SCC), which carries a poor prognosis, thus making a definitive diagnosis, no matter what size, essential. Here, we present a case of CHFC as well as a comprehensive literature review. Given these data, we propose an algorithm for definitive diagnosis.

## 1. Introduction

Ciliated hepatic foregut cyst (CHFC) is a rare benign cyst of the liver, derived from the embryonic foregut epithelium [1]. CHFCs are typically asymptomatic; however, some present with nonspecific abdominal symptoms or symptoms due to biliary obstruction [2]. Most cases are discovered incidentally, commensurate with the increase in abdominal imaging [3, 4], making it difficult to estimate the true prevalence; however, there are 70 reported cases. Imaging modalities such as ultrasound (US), computed tomography (CT), and magnetic resonance imaging (MRI) are insufficient for a definitive diagnosis due to nonspecific and variable imaging features noted correlating to benign and malignant pathologies [5–7]. Therefore, a definitive diagnosis of CHFC requires histologic confirmation [8] of ciliated pseudostratified columnar epithelium surrounded by a subepithelial connective tissue layer, a smooth muscle layer, and an outer fibrous capsule [1].

While typically benign, malignant transformation into squamous cell carcinoma (SCC) has been noted, particularly for large lesions (mean 10 cm) [3, 9]. As transformation portends a poor prognosis, early detection is essential [10]. The combination of nonspecific radiologic findings and the malignant potential highlight the need for a definitive diagnosis, most often achieved by surgical resection.

We present a case of CHFC and a comprehensive literature review and propose an algorithm for definitive diagnosis.

## 2. Case Report

A 75-year-old male underwent screening for lung cancer due to a history of tobacco use and a family history of lung cancer. Multiple, bilateral lung nodules, 9 mm and 5 mm, were noted on the left upper lobe; also noted was a 5 mm nodule on the right middle lobe and other smaller nodules. He was referred to pulmonology due to the size (≥8 cm) of the largest nodule and a new nodule (≥6 cm). A three-month follow-up by CT was recommended. Three-month follow-up revealed stable nodules classified as Lung-RADS 2. A one-year follow-up by CT was recommended. The one-year follow-up revealed multiple nodules of increased size on the left side, (Lung-RADS 4b). Bronchoscopy with bronchoalveolar lavage, endobronchial ultrasound, and needle aspiration obtained biopsies deemed negative for malignancy. A three-month follow-up was again recommended, noting growth of right and left lobe nodules. A PET scan showed bilateral hypermetabolic lung nodules; a CT-guided core needle biopsy of the right upper lobe nodule revealed a well-differentiated adenocarcinoma with an immunohistochemistry (IHC) profile, suggestive of GI or pancreaticobiliary origin (+CDX-2, + CK20, + CK7, − Napsin A, and − TTF1). A CT of the abdomen and pelvis was ordered to screen for a potential primary.

**Figure 1:**
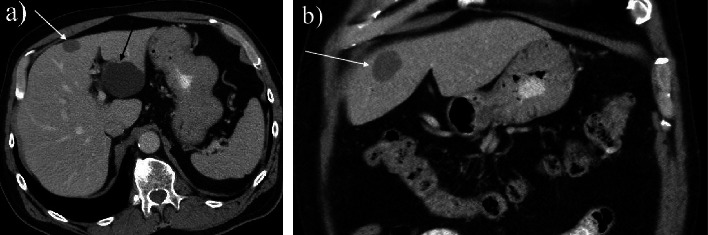
Intravenous contrast enhanced CT examination of the abdomen (a) axial (b) coronal demonstrating segment 4 hepatic lesion (white arrow) that has slightly increased and heterogeneous attenuation in comparison with segment 2/3 hepatic simple cyst (black arrow).

**Figure 2:**
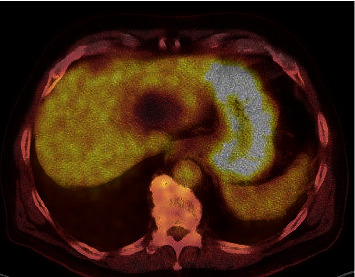
Axial CT fused image of FDG PET examination demonstrating no evidence of abnormal metabolic activity within segment 4 hepatic lesion.

**Figure 3:**
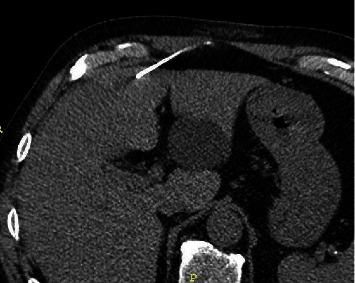
CT guided core biopsy of segment 4 hepatic lesion.

**Figure 4:**
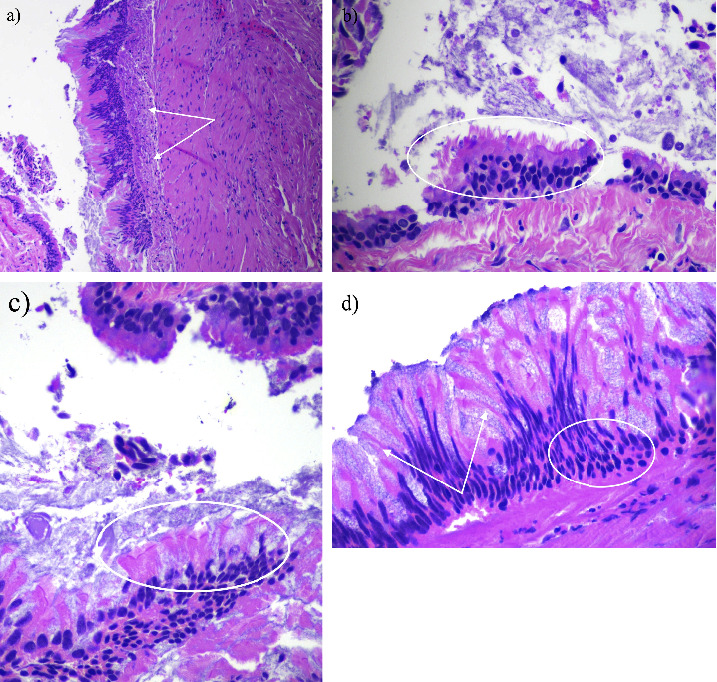
Ciliated hepatic foregut cyst histology. (a) Low power H&E showing pseudostratified columnar epithelium, with underlying subepithelial connective tissue and loose lamina propria (arrows). (b) High-power H&E showing ciliated pseudostratified columnar epithelium (example circled). (c) High-power H&E showing ciliated pseudostratified columnar epithelium with the overlying epithelium demonstrating admixed goblet cells (example circled). (d) High-powered H&E showing pseudostratified columnar epithelium (circled) with an abundance of admixed goblet cells (arrows).

CT of the abdomen (Figures 1(a) and 1(b)) revealed a simple benign cystic lesion in the left lateral hepatic lobe (segments 2/3) and an additional indeterminant nonenhancing, hypoattenuating lesion (48 HU) measuring 22 mm in seg 4B. Based on the level of attenuation, this lesion had a possibility of being solid. Further evaluation of this lesion by multiphase CT (Figure 2) revealed a nonenhancing, low-attenuating lesion located in seg 4B of the liver measuring 26 × 21 × 15 mm. In addition, intrahepatic ductal dilation was noted in segment 5, prompting concern for intrahepatic cholangiocarcinoma. Serum level of CA19-9 was within normal range while CEA was elevated (7.6 ng/mL; reference range 0.0–3.0 ng/mL). A CT-guided core biopsy of the liver lesion (Figure 3) was completed, revealing benign hepatocellular parenchyma without atypical change. As cholangiocarcinoma was still in the differential, a robotic partial hepatectomy was completed utilizing intraoperative ultrasound. A cystic lesion was identified in segment 4A/B and removed. Histology revealed a cyst surrounded by a fibrous capsule, well demarcated from adjacent unremarkable hepatic parenchyma. The cyst was noted to have an epithelial lining with varying complexity (Figures 4(a) and 4(b)), with portions lined by simple ciliated epithelium with abundant goblet cells (Figures 4(c) and 4(d)). No areas of dysplasia or malignancy were identified. The patient had an unremarkable surgical recovery and was discharged on day 3. CEA continued to be elevated postoperatively in the presence of the lung adenocarcinoma. All follow-up and treatment were focused on the adenocarcinoma of the lung with an unknown primary with no further concern regarding the liver.

## 3. Discussion

CHFC is a rare, typically benign liver cyst lined by ciliated pseudostratified columnar epithelium surrounded by subepithelial connective tissue, smooth muscle, and an outer fibrous layer. Differential diagnoses include simple hepatic cyst, parasitic cyst, epidermoid cyst, pyogenic abscess, intrahepatic choledochal cyst, hypovascular solid tumor, hepatobiliary cystadenoma, and cystadenocarcinoma [11]. Given this, a definitive diagnostic strategy is essential.

To facilitate this, a comprehensive review of the literature on CHFC was completed (Table 1) [12–49]. The mean age was 47 years (median 50; range 0–79) thus making our patient one of the oldest reported; two reports were prenatal diagnoses [31, 35]. CHFCs are mostly asymptomatic; however, symptomatic cases were not uncommon (43% of cases), primarily abdominal pain (36/37). In several cases, CHFC compressed hepatic ductal structures resulting in jaundice [2, 17, 18, 37, 46, 50]. The size of symptomatic cysts (median 6 cm, IQR 5.45; one outlier at 130 cm [49]) was significantly larger than the size of asymptomatic cysts (median 3 cm, IQR 1.9, *p* = 0.01). In our patient, the cyst was asymptomatic and discovered incidentally.

The most common location for CHFC includes segment 4 (48/78; 62%), with secondary locations including the anterior segment of the right lobe (segments 5 and 8; 19/78; 24%) or adjacent to biliary structures, including the gallbladder and biliary bifurcation, and vascular structures of the portal triad with three located within the gallbladder fossa [2, 14, 25, 27, 34, 45, 46, 48].

On imaging, the CHFCs were mainly unilocular (46/50; 92%), consistent with our patient. Imaging characteristics of CHFCs vary with T1 MRI but are mostly hyperintense (8/14; 57%); less commonly, CHFCs are hypointense (3/14; 21%) and isointense (3/14; 19%). On T2 MRI, they were almost always hyperintense (24/24; 100%) and never hypointense. CT imaging often notes a hypoattenuating lesion (90%; 36/40) and occasionally a hyperattenuating mass (4/40, 10%). For those cases that reported Hounsfield units (HU; *n* = 10), the median attenuation for hypoattenuating lesions was 54 HU (IQR 14.25) and mean attenuation was 53.2 HU (SD 6.18) [4, 6, 14, 19, 24, 26, 51]. In hyperattenuating lesions, attenuation was 80 HU [16, 51]. Outer rim enhancement was reported in two cases [31, 37]. MRI was rarely employed [31, 39, 41]. On ultrasound, most CHFCs were hypoechoic (94%; 30/32), similar to simple hepatic cysts; however, hepatic cysts demonstrate attenuation between 0–10 HU [52], whereas CHFCs produce a higher level of attenuation, as in our case. Thus, imaging alone cannot be reliably used to diagnose CHFCs.

Levels of serum AFP were normal in all CHFC cases tested (*n* = 10), and CEA was normal in most (11/12; 92%) [16]. In our patient, the elevation of CEA was attributed to the presence of lung adenocarcinoma, since it remained elevated after CHFC resection. CA19-9 is also not useful for a definitive diagnosis, as it was elevated in about a third of the tested cases (5/16; 31%).

Histology provides the definitive diagnosis through pathologic evaluation, preferably from a biopsy of the CHFC wall (48/48). While needle core biopsy produced a diagnostic sample in two cases, it was not successful in our case (2/3, 66%). FNA was diagnostic in 62% of cases (8/13), revealing ciliated epithelial cells in the aspirate.

Previous reports have suggested that the transformation of CHFC to SCC was a rare occurrence; however, SCC was present in 9% of reports (6/70; 9%), and squamous metaplasia without SCC was present in 6% of cases (4/70). The presence of squamous transformation was associated with a larger size (median 9 cm, IQR 5.25) than CHFC without transformation (median 3, IQR 3.98; *p* < 0.01) [3].

While imaging cannot definitively diagnose CHFCs, imaging characteristics may indicate the presence of a malignant or metaplastic process due to the disruption of typical cystic architecture. Out of five cases of SCC in which imaging features were described, atypical characteristics noted were as follows: a malignant cyst with wall irregularity on MRI [23], SCC with a region of calcification and a mural nodule on CT [47], and squamous metaplasia with calcification [29]. While solid wall components have been identified in benign cases, calcifications have only been reported in squamous transformation [8, 9]; heterogeneity in a metaplastic [39] and a malignant cyst [40] have also been described. Our report notes heterogeneity on CT, which has been reported in both benign and malignant cases [11, 22]. But some cases with squamous transformation do not exhibit any abnormalities on imaging, highlighting the limited use in definitively identifying malignancy. While all cases of SCC were resected, the transformation of CHFC to SCC was associated with poor outcomes [38], with 67% of reports resulting in metastatic spread or death (2–10 months) [18, 22, 41, 47].

In total, in all but three reported cases, patients underwent resection of the CHFC (66/69, 96%). Of note, laparoscopic resection is feasible, given that the cyst wall is thick enough to be excised from the hepatic parenchyma and was noted in eight reports [49]; one report also noted robotic resection [48]. Overall, outcome reporting was limited.

**Figure 5:**
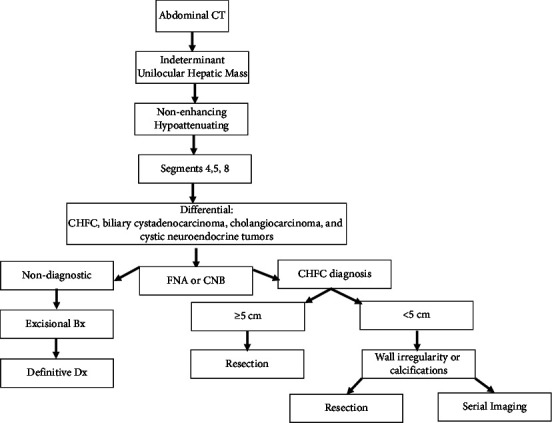
Diagnostic algorithm. Utilization of imaging and biopsy (CNB or FNA) to definitively diagnose and manage CHFC. Of note: incidental findings of potential CHFC during surgery for other entities would require further evaluation of the lesion to include imaging and classification to obtain a definitive diagnosis.

Given these data, we have developed an algorithm to assist in the definitive diagnosis of CHFC (Figure 5). In an indeterminant unilocular hepatic mass, especially with a nonenhancing, hypoattenuating lesion (40–60 HU) in segments 4, 5, or 8, FNA or core needle biopsy should be completed due to their noninvasive nature and potential for a diagnostic sample. If the samples are nondiagnostic, an excisional biopsy should be carried out to establish a diagnosis. If FNA or core biopsy confirms the diagnosis of CHFC, resection of CHFC ≥3 cm is warranted based on the positive association between the size and squamous transformation and the poor prognosis of malignant transformation. For cysts <3 cm, resection is warranted if atypical features such as wall irregularity or calcifications have been noted on imaging. In the absence of abnormal imaging, serial imaging follow-up is recommended to monitor growth or changes in the appearance of the cyst; symptomatic cysts warrant resection regardless of the size.

## 4. Conclusion

Besides biopsy, no single diagnostic feature of CHFC is highly specific or sensitive. However, the probability of squamous transformation in CHFC cannot be ignored, warranting an intentional and diligent treatment strategy. An indeterminant, unilocular nonenhancing, hypodense lesion should raise suspicion for CHFC, especially when located in the medial segment of the left hepatic lobe or anterior segment of the right lobe (segments 4, 5, and 8). These lesions should first be biopsied with FNA or core needle biopsy. If nondiagnostic, resect the lesion for a definitive diagnosis. If CHFC is diagnosed, determine the size of the cyst. If the cyst size is greater than/equal to 3 cm, resect due to the risk of malignancy. If it is less than 3 cm, monitor with serial imaging for growth or changes in appearance. If it is less than 3 cm but has atypical imaging features (e.g., wall nodule, irregularity, or calcifications), resect due to the risk of malignancy.

## Figures and Tables

**Table 1:** Comprehensive literature review^*∗*^.

Author	Year	Age	Gender	Symptoms	Location	Size (cm)	Imaging	Tumor marker	Biopsy	Squamous transformation	Treatment	Outcome
Dardik	1964	69	F	Abd pain, jaundice	R Lobe	9			Excision: diagnostic	Not present	Resection	
Wheeler	1984	69	M	Asymptomatic	Ant seg R lobe	2.5	Unilocular		Excision: diagnostic	Not present	Resection	
Kadoya	1990	41	M	Asymptomatic	Seg 4	2	Nonenhancing, hypoechoic (US), hypodense (CT), hypointense (SE 500/20). Hyperintense (SE 2500/25, SE 2500/80)		Excision: diagnostic	Not present	Resection	
Kadoya	1990	56	F	Asymptomatic	Seg 4	3	Unilocular, nonenhancing, hypoechoic (US), hyperdense (CT)		Excision: diagnostic	Not present	Resection	
Kadoya	1990	69	M	Abd pain	Seg 4	2	Nonenhancing, hypoechoic (US), hypodense (CT)		FNA: non-diagnostic	Not present		
									Partial wall: diagnostic			
Kimura	1990	67	M	Asymptomatic	Seg 4	3.5	Unilocular, nonenhancing, hypoechoic (US), hyperdense (CT), hyperintense (T1, T2)		Excision: diagnostic	Not present	Resection	Uncomplicated
Abe	1994	57	M	Asymptomatic	Seg 4	1.9	Unilocular, nonenhancing, hypoechoic (US), hypodense (CT), hypointense (T1), hyperintense (T2)		Excision: diagnostic	Not present	Resection	
Zaman	1995	35	F	Asymptomatic	Seg 4	3	Nonenhancing, hypodense (CT)		FNA: diagnostic	Not present		
Carnicer	1996	5	F	Abd discomfort	Between portal vein & gallbladder	2	Unilocular, nonenhancing, hypoechoic (US), hypodense (CT), isointense (T1), hyperintense (T2)		FNA: diagnostic	Not present	Observation	Resolved symptoms
Hornstein	1996	38	M	Abd pain	Ant seg R lobe	3	Hypoechoic (US)		FNA: diagnostic	Not present	Resection	
Hornstein	1996	53	F	Asymptomatic	Ant seg R lobe	3	Hypodense (CT)		FNA: diagnostic	Not present		
Hornstein	1996	69	M	Asymptomatic	Ant seg R lobe	3.5	Hypodense (CT)		FNA: diagnostic	Not present	Resection	
Murakami	1996	63	F	Asymptomatic	Seg 4	2.2	Nonenhancing, hypoechoic (US), hyperdense (CT), hyperintense (T2)	Normal AFP. Elevated CEA, CA19-9	FNA: non-diagnostic	Not present	Resection	
									Excision: diagnostic			
Harty	1998	17	F	Abd discomfort, jaundice, splenomegaly	R Lobe	8	Hypoechoic (US), hyperintense (T1, T2)			Squamous metaplasia		
Vick (a)	1999	51	M	Abd pain	R Lobe	12			Excision: diagnostic	SCC	Resection	Death
Vick (b)	1999	14	F	Jaundice		6	Multilocular			Not present		
Vick (b)	1999	45	M	Asymptomatic	Seg 4	3	Unilocular			Not present		
Vick (b)	1999	48	M	Asymptomatic	R Lobe		Unilocular			Not present		
Vick (b)	1999	59	F	Asymptomatic		3	Unilocular			Not present		
Vick (b)	1999	60	F	Asymptomatic		1.1	Unilocular			Not present		
Vick (b)	1999	61	M	Asymptomatic	Ant seg R lobe	2.5	Unilocular			Not present		
Kwak	2000	69	M	Asymptomatic	Seg 4	2.5	Unilocular, nonenhancing, hypodense (CT)		Excision: diagnostic	Not present	Resection	
Hirata	2001	53	M	Asymptomatic	Seg 4	4	Unilocular, nonenhancing, anechoic (US), hypodense (CT), hyperintense (T1, T2)		FNA: non-diagnostic	Not present		
									Excision: diagnostic			
Bogner	2002	55	M	Asymptomatic	R Lobe	2.5			Excision: diagnostic	Not present	Resection	
Furlanetto	2002	21	M	Abd pain, weight loss	Seg 5, 6	10	Polyseptated, hypoechoic (US), hypodense (CT)	Normal AFP, CEA, CA19-9	FNA: non-diagnostic	SCC	Resection, chemotherapy	Death
									Excision: diagnostic			
Lajarte-Thirouard	2002	40	F	Abd pain	Seg 5	13	Irregular posterior wall, nonenhancing, hypoechoic (US), hypodense (CT), hyperintense (T1, T2)		Excision: diagnostic	SCC	Resection	
Del Poggio	2003	58	F	Asymptomatic	Seg 4	2	Unilocular, nonenhancing, hypoechoic with hyperechoic spots (US), hypodense (CT), hyperintense (T2)		FNA: diagnostic	Not present	Observation	
Jakowski	2004	40	F	Abd pain, N/V	Seg 4	2.1	Unilocular, hypodense (CT), hypointense (T1), hyperintense (T2)		Excision: diagnostic	Not present	Resection	
Momin	2004	68	F	Asymptomatic	Seg 2, 3	3.8	Unilocular, nonenhancing, “solid appearing” (US), hypodense (CT), hypointense (MRV)		Excision: diagnostic	Not present	Resection	
Fang	2005	30	M	Abd discomfort	Seg 4	4	Unilocular, nonenhancing, hypodense (CT), isointense (T1), hyperintense (T2)		Excision: diagnostic	Not present	Resection	
Rodriguez	2005	20	F	Abd pain, anorexia	Gallbladder fossa		Unilocular, hyperintense (T2)		Excision: diagnostic	Not present	Resection	
Kang	2006	56	M	Asymptomatic	Seg 4	4.5	Unilocular, nonenhancing, hypodense (CT), hyperintense (T2)	Normal CEA, CA19-9		Not present	Resection	Uncomplicated
Ben Mena	2006	31	F	Abd pain	Seg 4	6	Unilocular, nonenhancing, hypoechoic (US), hyperdense (CT)	Normal AFP, CEA, CA19-9	Excision: diagnostic	Squamous metaplasia	Resection	
Straus	2006	63	M	Asymptomatic	Seg 4	1.5	Nonenhancing, hyperdense (CT)		Excision: diagnostic	Not present	Resection	Alive
Stringer	2006	0	M	Abd distension	Seg 5, 8	10	Unilocular, rim enhancing, hypoechoic (US), hypodense (CT), “cystic lesion” (MRI)		Excision: diagnostic	Squamous metaplasia	Resection	L hepatic duct necrosis
Kaplan	2007	68	M	Asymptomatic	Seg 4	3	Unilocular, nonenhancing, hypoechoic (US), hypodense (CT), hyperintense (T1, T2)	Normal AFP. Elevated CA19-9	FNA: diagnostic	Not present		
Young	2007	16	F	Abd pain, anorexia	Ant seg R lobe	6	Multilocular, hyperintense (T2)		FNA: non-diagnostic	Not present	Resection	
									Excision: diagnostic			
Shaw	2007	50	F	Abd pain	Porta Hepatis	8	Unilocular, hypodense (CT), hyperintense (T2)	Normal CA19-9	Excision: diagnostic	Not present	Resection	Uncomplicated
Betalli	2008	0	F	Asymptomatic	Seg 4	5	Unilocular, nonenhancing, hypoechoic (US), isointense (T1), hyperintense (T2)		Excision: diagnostic	Not present	Resection	
Geramizadeh	2008	25	M	Abd pain	Seg 8	2.7	Hypoechoic (US)			Not present	Resection	Uncomplicated
Kiyochi	2008	69	F	Abd pain, jaundice	Seg 4	2.5	Unilocular, rim enhancing, hypoechoic (US), hypodense (CT), hypointense (T1), hyperintense (T2)	Elevated CA19-9	Excision: diagnostic	Not present	Resection	
Sharma	2008	28	F	Asymptomatic	Seg 4	5	Solid component, nonenhancing, hypoechoic (US), hypodense (CT), hyperintense (T1, T2)	Normal AFP, CEA, CA19-9	Excision: diagnostic	Not present	Resection	
Zhang	2009	60	F	Abd fullness	Seg 4	7	Hypoechoic (US)	Normal AFP, CEA, CA19-9	Excision: diagnostic	SCC	Resection	Uncomplicated
Zaydfudim	2010	17	F	Abd pain, nausea	Seg 4	6.5	Hypoechoic (US), hypodense (CT)	Normal AFP	Needle core: diagnostic	Not present	Resection	Uncomplicated
Ambe	2012	42	M	Abd pain	Seg 4	7	Unilocular, hypodense (CT)		Excision: diagnostic	Not present	Resection	
Feernandez-Acenero	2012	35	F	Abd pain	Seg IV, V	7	US, CT	CK-19+; CA 19−9+: EMA+; TTF-1+			Resection	Alive
Feernandez-Acenero	2012	33	M	Asymptomatic	Seg IV	6.5	US; hyperintense (MRI)	CK-19+; CA 19−9+: EMA+; TTF-1+			Resection	Alive
Khoddami	2013	3.5	M	Abd pain	Seg 4	3.7	Nonenhancing, hypoechoic (US), hypodense (CT)		Excision: diagnostic	Not present	Resection	
Khoddami	2013	3.5	M	Asymptomatic	R Lobe	3.6	Nonenhancing, heterogeneous, hyperechoic (US), hypodense (CT)		Excision: diagnostic	Squamous metaplasia	Resection	
Wilson	2013	34	M	Asymptomatic	Seg 4, 5, 8	14	Nonenhancing, hypodense (CT), hypointense and heterogeneous (MRI)	Normal AFP, CEA, CA19-9	Excision: diagnostic	SCC	Resection, TACE, systemic chemotherapy after recurrence	Recurrence and metastasis
Ben Ari	2014	45	M	Abd pain	Seg 4	6.2	Multilocular, nonenhancing, hypoechoic (US), hypodense (CT)	Normal AFP, CEA, CA19-9	FNA: diagnostic	Not present	Resection	
									Excision: diagnostic			
Saravanan	2014	32	F	Abd pain	Seg 8	10	Nonenhancing, hypoechoic (US), hypodense (CT)	Normal CA19-9	Excision: diagnostic	Not present	Resection	
Bishop	2015	42	F	Abd discomfort	Porta Hepatis	8	Unilocular	Normal CEA, CA19-9	Excision: diagnostic	Not present	Resection	
Bishop	2015	46	F	Asymptomatic	R Liver	1	Unilocular		Needle core: diagnostic	Not present	Observation	Alive
Bishop	2015	50	F	Asymptomatic		10	Unilocular		Partial wall: diagnostic	Not present	Partial resection	Alive
Bishop	2015	58	F	Asymptomatic	Seg 4	0.7	Unilocular		Excision: diagnostic	Not present	Resection	
Bishop	2015	66	M	Abd pain, N/V, jaundice	Seg 4	17	Unilocular	Normal CEA. Elevated CA19-9	Excision: diagnostic	Not present	Resection	
Bishop	2015	67	M	Asymptomatic	Porta hepatis	6.5	Unilocular	Normal CEA, CA19-9	Excision: diagnostic	Not present	Resection	Alive
Bruns	2015	4	M	Asymptomatic	Seg 7	7.4	Hypoechoic (US), hyperintense with enhancing septations (T2)	Normal AFP	Excision: diagnostic	Not present	Resection	Uncomplicated
Grizzi	2015	33	M	Asymptomatic	L Lobe	6.5	Hypoechoic (US), hyperintense (T2)			Not present	Resection	
Grizzi	2015	35	F	Abd discomfort	Seg 4, 5	7	Hypoechoic (US), hypodense (CT)			Not present	Resection	
Grizzi	2015	45	M	Abd pain	Seg 4	2	Hypoechoic (US)			Not present	Resection	
Grizzi	2015	63	F	Asymptomatic			Unilocular, hypodense (CT)			Not present	Resection	
Beteddini	2016	33	F	Abd pain, vomiting	Triangle of Calot	1.5	Unilocular		Excision: diagnostic	Not present	Resection	
Cottreau	2016	57	F	Abd pain, jaundice	Common hepatic duct	1.7	Unilocular, hypoechoic (US), hyperintense (T2)		Excision: diagnostic	Not present	Resection	Uncomplicated
Ansari-Gilani†	2017	52	M	Abd pain	Seg 4	2	Hypodense (CT), hyperintense (T2)		Unspecified	Not present		
Ansari-Gilani†	2017	62	F	Asymptomatic	Seg 4	1.5	Nonenhancing, hypodense (CT), hyperintense (T1)		Unspecified	Not present		
Ansari-Gilani^†^	2017	68	M	Asymptomatic	Seg 4		Nonenhancing, hypodense (CT), hyperintense (T1, T2)		Unspecified	Not present		
Enke	2019	54	M	Asymptomatic	Seg 4	2.7	Solid component, nonenhancing, hypodense (CT), hyperintense (T2)		Excision: diagnostic	Not present	Resection	Uncomplicated
Itose	2020	50	F	Abd pain	Seg 4	4	Solid component, calcification, hypodense (CT)	Normal AFP, CEA. Elevated CA19-9	Excision: diagnostic	SCC	Resection, chemotherapy	Recurrence and metastasis; alive at 30 months
Ziogas	2020	39	M	Incidental	4B	2.5	Unilocular				Lap wedge resection/cyst unroofing	1 LOS
Ziogas	2020	73	F	Incidental	7	2.9	Unilocular				Wedge resection	4 LOS
Ziogas	2020	64	F	Incidental	4A	2	Unilocular				Central Liver wedge resection	7 LOS
Ziogas	2020	49	M	Incidental	4B	2.5	Unilocular				Lap wedge resection	2 LOS
Ziogas	2020	61	F	Incidental	4A	2.4	Unilocular				Rob wedge resection	3 LOS
Ziogas	2020	17	M	Incidental	4A	1.5	Unilocular				Lap wedge resection/cyst unroofing	0 LOS
Ziogas	2020	63	M	Epigastric pain	4A	2	Unilocular				Lap wedge resection	2 LOS
Ziogas	2020	62	M	Incidental	Falciform ligament	0.8	Unilocular				Lap wedge resection	2 LOS
Ziogas	2020	58	M	Incidental	4A	3	Multilocular				Lap wedge resection/cyst unroofing	1 LOS
Ziogas	2020	47	M	Incidental	4	1.3	Unilocular				Lap wedge resection	1 LOS
Ziogas	2020	67	M	Incidental	Falciform ligament	2	Unilocular				Hepatectomy for liver transplant	83 LOS
Ziogas	2020	79	M	Abd pain (lower)	4	3	Unilocular				Wedge resection	18 LOS
Ziogas	2020	78	M	Abd pain (right side)	4A-8	3.5	Unilocular				Wedge resection/cyst unroofing	110 LOS
Seyed-alagheband	2023	41	F	Abd pain; palpable mass	Seg 4	130	Subhepatic cyst with internal septations (US and CT)	Normal LFTs	No	Not present	Lap resection	Uncomplicated 1.5 years
Kato	2023	75	M	Asymptomatic	Seg 4	2.6	Unilocular, nonenhancing, hypodense (CT)	Normal AFP. Elevated CEA	Excision: diagnostic	Not present	Resection	Uncomplicated

^*∗*^All reports were in English and able to be procured by our institution; grayed boxes indicate that the report did not provide that information; age = age at diagnosis; ^†^author reports a biopsy was conducted by did not specify the type; Vick et al. reports a case of SCC (a) and a review of 6 cases (b), both published in the same year; “nausea and vomiting” abbreviated with “N/V;” “abdominal” abbreviated with “abd;” “left” and “right” abbreviated with “L” and “R,” respectively; “anterior” abbreviated with “ant;” “segment” abbreviated with “seg.”

## Data Availability

The data used to support the findings of this study are restricted by the Prisma Health SC IRB in order to protect patient privacy and be in compliance with HIPAA. Data are available from Angela Wishon, VP-Research Compliance (angela.wishon@prismahealth.org) for researchers who meet the criteria for access to confidential data with an appropriate Data Use Agreement.
